# Quality of working life of cancer survivors: development of a cancer-specific questionnaire

**DOI:** 10.1007/s11764-015-0485-4

**Published:** 2015-09-14

**Authors:** Merel de Jong, Sietske J. Tamminga, Angela G. E. M. de Boer, Monique H. W. Frings-Dresen

**Affiliations:** Coronel Institute of Occupational Health, Academic Medical Center, University of Amsterdam, P.O. Box 22660, 1100 DD Amsterdam, The Netherlands

**Keywords:** Quality of working life, Cancer survivors, Questionnaire, Work continuation

## Abstract

**Purpose:**

The aim of this study was to generate, and select quality of working life issues for the development of an initial version of the Quality of Working Life Questionnaire for Cancer Survivors (QWLQ-CS).

**Methods:**

Quality of working life issues were generated through focus groups with cancer survivors and oncological occupational physicians, and interviews with employers, supervisors, and organization officers. A selection of these quality of working life issues was made based on relevance and importance by conducting an online questionnaire among the cancer survivors and oncological occupational physicians. Researchers formulated the issues into items for the QWLQ-CS.

**Results:**

A total of 24 cancer survivors, six oncological occupational physicians and 11 employers, supervisors, and organization officers participated. The 222 quality of working life issues identified through the focus groups, interviews, and literature were converted into an online questionnaire. Cancer survivors (*N* = 20) found 44 issues not relevant or important with respect to their quality of working life. The researchers reviewed the remaining 178 issues and formulated them into 102 items classified by five categories: work perception, job characteristics, the social structure and environment, organizational characteristics, and the effect of the disease and treatment.

**Conclusions:**

The initial version of the QWLQ-CS exists out of 102 items which cover the experiences and perceptions of cancer survivors in the work environment. All items were indicated by working cancer survivors as relevant and important.

**Implications for cancer survivors:**

This initial version of the QWLQ-CS may increase awareness of the potential problems or emotional difficulties working cancer survivors face during the work continuation process.

## Introduction

As cancer is developing into a chronic disease because of enhanced treatments and life prognosis, the number of people that continue living with a diagnosis of cancer is rising [1], and a substantial proportion of cancer survivors return to work after their diagnosis. Consequently, the number of working cancer survivors will increase. In addition, higher prevalence rates of cancer in older workers and the raising of the retirement age also contribute to this trend [2–4].

Work is important to cancer survivors; it signifies a return to normalcy and control, and it contributes to quality of life [5, 6]. Nevertheless, cancer survivors often experience changes when returning to work, for instance in work-based social support, altered work priorities, and impaired work ability [7]. These experiences result in problems, such as fatigue, loss of confidence, or being overprotected by the employer [8], and might interfere with the return-to-work (RTW) or work continuation process. The problems are perceived as barriers, such as a lack of understanding from the work environment that hampers a post-RTW phase [9]. In short, cancer survivors face many challenges in their working life.

Previously, the working life of cancer survivors was studied by measuring for example work participation, productivity, and work loss [10–12]. This type of research encompasses quantitative outcomes that can be measured in an objective manner, but it does not include work-related problems experienced by cancer survivors at work. How cancer survivors perceive work-related problems, or a subjective perspective on their working life, has not been a topic of interest in major research. Hence, the working life of cancer survivors is often evaluated in a quantitative manner rather than a qualitative one, and the subjective work-related problems mentioned above are not taken into account.

The importance of approaching working life in a subjective manner is demonstrated by studies that associated high Quality of Working Life (QWL) with increased job satisfaction and lower levels of turnover intentions in “healthy” employees [13, 14]. Most research on QWL has been performed solely among “healthy employees”. Previous studies on QWL focused on individuals’ experiences in the job, organizational, and social environment [15–17]. Even the questionnaires used today for measuring QWL were developed for healthy employees [15, 16, 18, 19], or specific occupations, such as nurses [20]. However, these groups differ from the group of employed cancer survivors because of the different problems cancer survivors face in their work due to their health problems and treatment. For instance, the NIOSH Quality of Worklife module [18] includes items about physical and mental health, but not items about cognitive limitations that can result from chemotherapy and that can influence the QWL of cancer survivors. The same applies to the Quality of Working Life Systematic Inventory (QWLSI) [19] that consists of items relevant to QWL, yet does not incorporate the emotional impact of cancer on the meaning of work or its possible influence on the QWL of cancer survivors. In addition, the Brooks’ Quality of Nursing Work Life Survey [20] is developed for nurses, and is therefore less relevant for cancer survivors with different professional backgrounds. In sum, a new valid and reliable QWL questionnaire is required, specific to cancer survivors.

The aim of this study was to generate and select QWL issues for the development of an initial version of the Quality of Working Life Questionnaire for Cancer Survivors (QWLQ-CS). Our research questions are as follows:

1. What issues contribute to the QWL of cancer survivors?
2. What relevant and important issues should be selected for the initial version of the Quality of Working Life Questionnaire for Cancer Survivors (QWLQ-CS)?

## Methods

### Study design

The guidelines for developing Questionnaire Modules of the EORTC Quality of Life Group [21] are designed for developing new questionnaires modules, and therefore also useful in developing our Quality of Working Life Questionnaire for Cancer Survivors (QWLQ-CS). Based on the guidelines, this study is divided into two different parts. The objective of the first part was to generate QWL issues by means of a qualitative study, while the second part was aimed at making a selection of these QWL issues by conducting an online questionnaire. For the sake of explicit and comprehensive reporting, we based our qualitative study on the consolidated criteria for reporting qualitative research (COREQ) [22]. Although no guidelines exist for reporting the findings of the online questionnaire, we reported the study process thoroughly. The Medical Ethics Committee of the Academic Medical Center considered ethical approval for both parts of this study unnecessary (W13_196#13.17.0241).

### Part 1: Generation of QWL issues

#### Participants

Three groups participated, i.e., cancer survivors, healthcare professionals, and employers. Cancer survivors were recruited from an academic medical center in the Netherlands and purposively selected on the basis of their cancer diagnosis. The cancer survivors were approached by their attending specialist if they were: (1) diagnosed with either lymphoma, leukemia, prostate cancer, testicular cancer, or breast cancer; (2) between 18 and 63 years of age; (3) in paid employment or self-employed; and (4) treated in the medical center between 2000 and 2011. If they agreed to participate in the study, the first author (MdJ) contacted them by phone or email. The healthcare professionals in this study were occupational physicians specialized in oncology, and they were recruited through the Netherlands School of Public and Occupational Health (NSPOH). In order to participate, the oncological occupational physicians (OOP) had to be engaged in assisting cancer survivors with work, and thus had knowledge about the processes of return-to-work or work continuation. The OOP received an email containing information about the study, with the request to indicate if they wished to participate in the study. Employers, supervisors, and other organization officers were approached through: (1) a national project about cancer at the workplace; (2) newsletters; (3) social media; and (4) snowball sampling. Employers, supervisors, or other organization officers could participate if they had experience with one or multiple employees with cancer within their organization. The first author (MdJ) contacted them by phone or email if they agreed to participate. All the participants signed an informed consent form before the start of the focus groups or interviews. For their participation, they received a €25 voucher and they were able to declare their travel expenses.

#### Procedure

For the generation of QWL issues, we conducted focus groups as well as interviews. Three separate focus groups were held with cancer survivors, and one focus group with OOPs. The meeting was audio-recorded and moderated by the second author (ST), who is a female post-doc researcher with extensive experience on the topic of cancer and work, and in performing qualitative research. The first author (MdJ) developed a guide for the focus group, organized the meetings, and acted as the observer. In advance, participants were asked to fill in a questionnaire at home and bring that with them to the focus group. The questionnaire for cancer survivors contained demographic questions such as age, household composition, education and employment status, and questions about their disease. We asked the OOPs about their demographics and their experiences with employees with cancer.

The focus group started with a broad, open-ended question which allowed the moderator to explore the reported issues in more detail. Cancer survivors were asked: “What makes you happy at work, what makes you enjoy your work and allows you to continue your work?”. OOPs were asked: “What contributes to the QWL and work continuation of employees with cancer you assisted during you career?”. At the end of the focus group meeting with the OOPs, we presented a list with five QWL themes extracted from the literature [23] in order to identify missing QWL issues.

The semi-structured interviews with employers, supervisors, and organization officers were conducted by the first author (MdJ), who is a female PhD candidate and involved in the topic of cancer survivors, QWL, return-to-work, and work continuation. An interview guide was developed, and all interviews were audio-recorded. The interviewer started with an open question: “What contributes to the QWL and work continuation of employees with cancer you have assisted within this organization or during your career?”. Subsequently, the interviewer explored the reported issues in more detail. At the end, the participant was shown a list with QWL themes extracted from the literature in order to identify missing QWL issues [23]. This list contained the following QWL themes: (1) job characteristics; (2) social structure and environment; (3) organizational characteristics; (4) individual work perceptions; and (5) effect of the disease and treatment.

#### Analysis

The data from the interviews were transcribed verbatim and coded using the MAXQDA (Verbi Software, GmbH Marburg 2007) qualitative data analysis software package. The first author (MdJ) administered open codes to the interview data by adding labels that represented the text as closely as possible. The codes were subdivided into five QWL themes that we identified in advance through our systematic literature review on QWL issues [23]. To check for consistency and reliability, i.e., whether all codes about QWL were administered correctly, the second author (ST) labelled the codes of 10 % of the interview data. The authors compared the codes, and in the case of any disagreement, the codes were discussed and made definitive during a consensus meeting.

The notes that were made during the focus groups were completed with the audio records of those meetings. The first author (MdJ) processed the data in MAXQDA in the same way as the interview data. Again for consistency and reliability, the second author (ST) checked 10 % of the focus group data to see whether all notes about QWL issues were reported by MdJ correctly. She also labelled these codes and checked if they matched the labels that had already been applied by MdJ. The outcomes were discussed until consensus was reached. Data saturation was striven for by reviewing the reported issues and searching for new QWL issues in every new focus group and interview. We based the number of interviews and focus groups on these findings. After finishing encoding the QWL issues in MAXQDA, the classification of the coding tree was discussed by all authors, and consensus was reached. The participants did not provide feedback on the transcripts or findings.

### Part 2: Selection of QWL issues

#### Participants

All the participants from the qualitative study gave their permission for a follow-up study, resulting in July 2014 in a total of 27 cancer survivors and 13 experts (i.e., OOPs, employers, supervisors, and organizational officers) receiving a personal invitation by email containing the link to the online questionnaire. This online questionnaire was administered to 27 cancer survivors, 24 of whom had participated in the focus groups and three who had been interviewed as an employer but turned out to be cancer survivors themselves. One week after the invitations had been sent, the participants who had not yet opened the survey or completed it received a reminder. If they participated, they were offered a voucher of €20 as a token of appreciation and a newsletter showing the outcomes of the qualitative study.

#### Procedure

To construct the online questionnaire, we converted the coding tree from the qualitative study into a list with QWL issues. Subsequently, we combined these QWL issues with the QWL issues retrieved from our systematic literature review and classified all issues in the following categories: individual work perceptions, job characteristics, social structure and environment, organizational characteristics, and the effect of the disease and treatment [23]. Next, this list was used to design an online questionnaire with the online survey software Fluidsurveys (SurveyMonkey Europe, Ireland 2014). We created two sets of questionnaires: (1) for the cancer survivors about relevancy and importance of the items; and (2) for the experts about the importance of the items. The group of cancer survivors was asked to rate each QWL issue for relevance on a 4-point Likert scale (1 = no relevance and 4 = very relevant). Additionally, they were asked to identify five key issues which they found most important to their QWL and indicate with a single question whether they considered the list to be complete on a 4-point Likert scale (1 = not complete and 4 = very complete). The order in which the issues were presented differed per participant. The experts were solely asked to identify which five key issues they found most important for employees with cancer and to indicate whether the list was complete. The reason for composing two sets of questionnaires was because the items must be relevant and important to the sample when developing a questionnaire [24]. Only cancer survivors can indicate if an item is relevant to their QWL, while both cancer survivors and experts can score the importance of items.

#### Analysis

Using the responses from cancer survivors on the relevance of QWL issues, we calculated the median relevance scores of each QWL issue. Furthermore, we reviewed the QWL issues that were indicated as being most important by the three sources. In order to select issues for the provisional questionnaire, the authors based the following decision rules on the EORTC guidelines: (1) If the median on relevance of a QWL issue was ≥2, the issue was included; and 2) if the median on relevance of a QWL issue was <2, then an issue was only included if ≥25 % of the cancer survivors indicated the issue to be important AND ≥50 % of the experts together indicated the issue to be important. When a respondent found the list to be incomplete and suggested another QWL issue, the authors would discuss it and include it if another issue did not already comprise the content of that issue and was therefore important to add to the provisional questionnaire.

## Results

### Part 1: Generation of QWL issues

#### Focus group with OOPs

The focus group session consisting of OOPs took place in November 2013; it lasted 77 min and was held in an academic medical center in the Netherlands. This group consisted of six OOPs, half of whom were male (*N* = 3) and with a mean age of 58 years. Together with the employers, supervisors, and organizational officers, they are referred to as experts (Table 2). The OOPs had worked an average of 21 years (SD = 10) as an OOP and were all specialized in the field of oncology. While some (*N* = 3) had provided occupational support to approximately a total of 30 employees with cancer, others advised more than 500 employees with cancer on work in the course of their career.

#### Focus group with cancer survivors

The three focus group sessions with cancer survivors were organized between December 2013 and February 2014, also at an academic medical center in the Netherlands. The duration of the sessions varied between 60 and 81 min, as did the number of cancer survivors per meeting (*N* = 5–10). Seventy percent of the 24 cancer survivors were male (*N* = 17), and the majority were highly educated, carrying out a variety of occupations (Table 1). The mean age of the cancer survivors was 54 years (SD = 7). The participants were diagnosed between 2000 and 2012 with lymphoma (13 %), leukemia (29 %), prostate cancer (21 %), testicular cancer (8 %), and breast cancer (8 %). The remaining 8 % were diagnosed with Kahler’s disease and Langerhans cell histiocytosis. Three cancer survivors had more than one primary cancer diagnosis (13 %). The majority had undergone either surgery (38 %) or chemotherapy (50 %). In total, 20 cancer survivors had a permanent position, and four were self-employed. After being diagnosed with cancer, most cancer survivors returned to their full-time jobs (*N* = 17), a smaller number worked between 12 and 32 hours (*N* = 6), and one person was on 100 % sick leave after he had initially returned to work.

**Table 1:** Characteristics of the cancer survivors

		Focus groups		Online questionnaire	
		*N* = 24		*N* = 20	
Demographic characteristics					
Age (mean in years ± SD)		54 ± 7		54 ± 7	
		*N*	(%)	*N*	(%)
Gender—male		17	(70.8)	12	(60.0)
Marital status	Married/living together with a partner	17	(70.8)	14	(70.0)
	Working partner	15	(88.2)	12	(60.0)
	Sole breadwinner	10	(59.0)	7	(50.0)
	Single	3	(12.5)	2	(10.0)
	Divorced	4	(16.7)	4	(20.0)
Diagnosis	Lymphoma	3	(12.5)	3	(15.0)
	Leukemia	7	(29.2)	6	(30.0)
	Prostate cancer	5	(20.8)	4	(20.0)
	Testicular cancer	2	(8.3)	0	(0.0)
	Breast cancer	2	(8.3)	4	(20.0)
	Kahler’s disease	1	(4.2)	1	(5.0)
	Langerhans cell histiocytosis	1	(4.2)	1	(5.0)
	Multiple cancer diagnoses	3	(12.5)	1	(5.0)
Treatment	Surgery	5	(20.8)	3	(15.0)
	Chemotherapy	4	(16.7)	4	(20.0)
	Other (e.g., radiotherapy, stem cell transplant, hormone therapy)	2	(8.3)	2	(10.0)
	Combined treatment from the above	13	(54.2)	11	(45.8)
Work characteristics					
Education	Intermediate vocational education	4	(16.7)	2	(10.0)
	Secondary education	4	(16.7)	3	(15.0)
	Higher professional education	11	(45.8)	12	(60.0)
	Academic education	5	(20.8)	3	(15.0)
Work contract	Permanent position	20	(83.3)	10	(50.0)
	Self-employed	4	(16.7)	6	(30.0)
Contract hours	Full-time	17	(70.8)	12	(60.0)
	Part-time (12–32 h)	6	(25.0)	3	(15.0)
	Sick leave (100 %)	1	(4.2)	1	(5.0)
Occupations	Management^a^	6	(25.0)	5	(25.0)
	Clerical^b^	3	(12.5)	2	(10.0)
	Education^c^	4	(16.7)	3	(15.0)
	Health^d^	3	(12.5)	3	(15.0)
	Municipality^e^	2	(8.3)	1	(5.0)
	Other^f^	6	(25.0)	6	(30.0)

^a^e.g., CFO, Human Resources manager, Information Records manager, Program and Change manager, account/project manager, Quality manager, team leader

^b^e.g., administration/financial staff, accountant

^c^e.g., headmaster, teacher, care coordinator

^d^e.g., physician, occupational nurse, social worker

^e^e.g., civil servant, municipal management coordinator

^f^e.g., airport security, journalist/program maker, or self-employed with businesses such as administration/taxes, human support, and real estate agency

#### Semi-structured interviews with employers, supervisors, and organization officers

A total of 11 employers, supervisors, and organization officers were interviewed from November 2013 to March 2014. These semi-structured interviews took place at the participants’ workplace or at a nearby location, with no other people being present besides the interviewer and participant. The duration of the interviews varied between 35 and 65 minutes. Forty-five percent of the employers, supervisors, and organization officers were male (*N* = 6), and their mean age was 50 years (Table 2). These participants included officers from the Human Resource department, managers, and self-employed advisors or coaches specialized in cancer and return-to-work. Experiences among the participants differed; some employers supported one employee with cancer within their company, while others had supported approximately a hundred employees over the years.

**Table 2:** Characteristics of the experts

		Focus groups or interviews		Online questionnaire	
		*N* = 17		*N* = 9	
Demographic characteristics					
Age (mean in years ± SD)		52 ± 7		55 ± 7	
		N	(%)	(N)	(%)
Gender—male		8	(47.1)	5	(55.6)
Work characteristics					
Occupations	Management^a^	5	(29.4)	3	(33.3)
	Human Resources^b^	2	(11.8)	0	(0.0)
	Health^c^	8	(47.1)	6	(66.7)
	Other^d^	2	(11.8)	0	(0.0)
Number of employees supported	0–50	12	(70.6)	6	(66.7)
	50–100	1	(5.9)	0	(0.0)
	≥100	4	(23.5)	3	(33.3)

^a^e.g., Director, Health manager, team manager, headmaster in primary education

^b^e.g., HR advisor, HR manager

^c^e.g., Head nurse, occupational nurse, (oncological) occupational physicians

^d^e.g., Self-employed with businesses such as human support, coaching, massaging

#### QWL issues

The analysis of the focus groups and interviews revealed 308 issues that contributed to QWL of cancer survivors. These codes represented the issues from the focus groups and interviews as closely as possible. For instance, the OOPs considered the security at work provided by the employer important to QWL of employees with cancer. They mentioned the importance of the employees’ autonomy within their (work) lives, as one OOP stated: “They need to learn how to deal with things more autonomously and to set their boundaries”. The cancer survivors mentioned the flexibility within their work as a contributor to QWL and having “nice” colleagues. The meanings of work, being able to contribute and therefore mean something instead of being “the helpless patient”, were specified as important issues. One cancer survivor mentioned that his ability to put things in perspective had changed: “I say it once, and if it doesn’t happen and it happens in a different way…fine by me!”. The interviews with the employers yielded both similar but also different issues, such as the importance of a good relationship between the employee and their supervisor and open communication between these two actors. One manager mentioned a more negative situation, with the stigma of cancer resulting in a taboo at the workplace. Due to the confrontation with the illness of their supervisor, employees felt reluctant to keep in contact with him. He found this situation difficult to deal with: “He had expected the whole team to surround him like a warm blanket, but well…this was a male-dominated sector”.

### Part 2: Selection of QWL issues

#### Online questionnaire

**Fig. 1:**
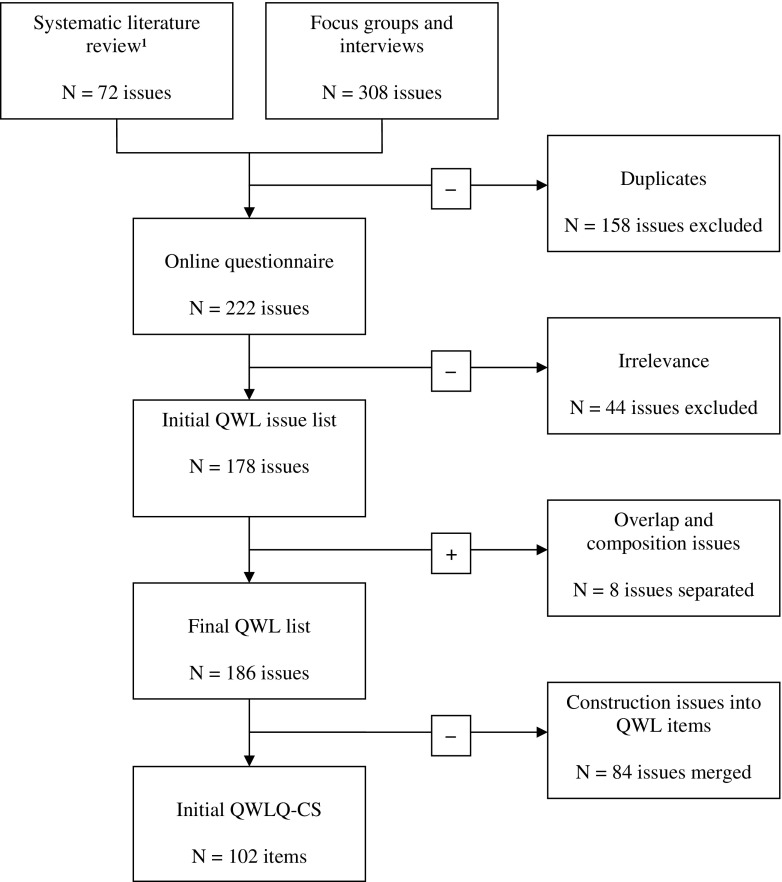
Flow chart of the selection of issues into items of the QWLQ-CS^1^ [23] de Jong et al., 2015

We prepared the online questionnaire by combining the coding tree of the focus groups and interviews with the 73 QWL issues of our systematic literature review (Fig. 1) [23]. After removing the duplicates, a total of 222 issues remained on the list. These issues were tested in the online questionnaire, which resulted in a slightly higher (74 %) response rate of the 20 cancer survivors, compared to the responses of nine experts (69 %). Both their demographics are displayed in Tables 1 and 2.

#### Relevance and importance of QWL issues

Of the 222 QWL issues, 44 issues scored a median of <2 which means that they were considered irrelevant to the QWL of the cancer survivors. None of the irrelevant issues were mentioned as being important by ≥25 % of the cancer survivors and ≥50 % of the OOPs and employers together. Of the cancer survivors, 60 % found the list “complete” and the remaining 40 % considered it to be “very complete”; they did not make any suggestions for other issues. One participant in the OOPs and employers group specified financial issues and cutbacks in salary as an additional issue. However, this issue had already been mentioned in the list. The other 89 % also found the list “complete” and “very complete”.

#### Selection of issues

After removing the irrelevant issues, the list with 178 issues was reviewed by the researchers. Some separate issues with the same meaning were combined; for instance, “workload” and “pressure of work” were combined into one issue “workload”. While reviewing the issues, the researchers also separated issues with different meanings, for example the issue “amount of self-confidence and self-esteem” was separated into two issues. This resulted in the final list containing eight more issues, making a total of 186 issues that were translated into items for the initial Quality of Working Life Questionnaire for Cancer Survivors (QWLQ-CS). Items that still overlapped were captured into one item, and those that used difficult or complex phrasing were rewritten using simpler wording. The researchers checked the items for clarity and specificity; for instance, the issue “open communication” was clarified as “clear communication with colleagues”. The total number of constructed items was 102, which were divided into three different sections in the initial QWLQ-CS. The first part contained items about work experiences in general, the second was about positive experiences, and the third section was about negative experiences at work. To provide a clear overview in the second and third sections, a subdivision was made of five categories from the literature review: individual work perceptions, job characteristics, social structure and environment, organizational characteristics, and the effect of the disease and treatment [23]. Issues about positive experiences were constructed into positively phrased items, and issues about negative experiences and limited functioning were negatively phrased. All items had to be answered within a 1-month time frame on a 4-point Likert scale (1 = disagree completely and 4 = agree completely). The answering option “not applicable” was also provided as some items were conditional. For instance, a participant who is self-employed cannot answer the item relating to his or her employer or colleagues. The items in the initial Quality of Working Life Questionnaire for Cancer Survivors (QWLQ-CS) are illustrated in Table 3.

**Table 3:** Items of the initial QWLQ-CS

Category	Phrased	Items
General	Positive	1.I am satisfied with my work
		2.I appreciate my work
		3.I have positive feelings about my work
		4.I enjoy doing my work
		5.I think my work is important
		6.I really want to work
		7.I think it is good to work
		8.I think it is necessary to work
		9.I think my work is meaningful because I am doing something useful
		10.I think my work gives me a purpose in life
		11.Because I work, I count in life
		12.I am suitable for my work
Work perceptions	Positive	13.I feel involved with the organization
		14.I feel connected to the organization
		15.I feel valuable for my colleagues and/or the organization
		16.I feel welcome at work
		17.I have a good work-life balance
		18.I have the feeling I can be myself
		19.I am self-confident in my work
		20.I am in charge at work
		21.I have self-esteem in my work
		22.I am able to develop my personal skills
	Negative	23.I feel uncertain about the future
		24.I am afraid the disease will return
		25.I think my work responsibilities are hard
		26.I think my work duties are hard
		27.I am afraid my job function will change
		28.I feel powerless
		29.I am afraid to make mistakes
		30.I feel vulnerable
		31.I feel helpless
Job characteristics	Positive	32.Work gives structure to my life
		33.I like my work activities
		34.I like my work duties
		35.I am satisfied with my work hours
		36.Years of service is important to me
		37.I am certain that I will keep my job
		38.I am satisfied about the fringe benefits of my work
		39.I am satisfied with my salary
		40.I am pleased to determine my work hours
		41.I am pleased to determine my work places
		42.I am satisfied with the adjustments in my work duties
		43.I am satisfied with the adjustments in my work hours
		44.I am satisfied with the facilities at my work
		45.My work circumstances are good
		46.I have a nice workplace
		47.My workplace is easily accessible
		48.My career opportunities are good
		49.I receive good further education and in-service-training
	Negative	50.I have to travel a long way to get to work
		51.I experience a high workload
Social structure and environment	Positive	52.The contact with my workplace is good
		53.I receive support from my social environment outside work
		54.I have a good relationship with my supervisor
		55.I receive support from my supervisor
		56.I have good relationships with my colleagues
		57.I receive support from my colleagues
		58.I receive support from my specialist and/or occupational physician
		59.I receive support from my buddy and/or confidential advisor
		60.I appreciate the fact that my specialist and/or occupational physician is giving me concrete information
		61.I have good social contacts with my customers/clients
		62.My clients cooperate with me
		63.I experience a positive atmosphere in my work environment
		64.I think that employees with a disease are treated well in my organization
		65.I experience a safe atmosphere in my work
		66.I feel they have faith in me at work
		67.I am taken seriously in my work
		68.I like to participate in social activities at my work
		69.I like to participate in social activities in my private life
		70.I am respected by my colleagues
		71.I am respected by my supervisor
		72.My colleagues understand my disease and its symptoms
		73.My supervisor understands my disease and its symptoms
		74.I disclose my disease and the current situation
		75.I communicate clearly with my colleagues
		76.I communicate clearly with my supervisor
		77.I communicate clearly with the Human Resources department
	Negative	78.My environment has high expectations of me
		79.My colleagues have high expectations of me
		80.My supervisor has high expectations of me
		81.My colleagues feel pity for me
		82.My occupational physician/Human Resources department have a negative attitude towards me
		83.I experience taboos about my disease at work
		84.I have negative confrontations with colleagues
Organizational structure	Positive	85.Departments communicate clearly about the adjustments in my work
		86.Within the organization, clear agreements are made concerning my work
		87.In my organization there are clear company regulations
		88.The organization is involved with me as an employee
	Negative	89.There is not much openness about the course of events in my organization
Effects of the disease and treatment	Positive	90.I am performing
		91.I am productive
		92.I am able to estimate whether I can perform
		93.I accept my disease and limitations
		94.I feel I am in charge of my (working) life
		95.I can resume my work activities
	Negative	96.I have difficulties recovering because of my symptoms and/or side effects
		97.I experience stress at work
		98.I am limited at my work because of my disease
		99.I have poor health with concentration/memory problems
		100.I have poor health with a shortage of energy/fatigue
		101.I am losing confidence in my own body
		102.I experience stress in my private life

## Discussion

The aim of this study was to generate and select QWL issues for the development of an initial version of the Quality of Working Life Questionnaire for Cancer Survivors (QWLQ-CS). In the first part, QWL issues were generated using qualitative methods, resulting in 222 QWL issues. In the second part, a total of 186 issues were selected based on relevance and importance using an online questionnaire and translated into 102 items for the initial version of the QWLQ-CS.

### Strengths and limitations

The development of the QWLQ-CS was based on the guidelines for developing Questionnaire Modules of the EORTC Quality of Life Group [21]. We followed these predefined steps in a systematic manner and used different methods for the generation of QWL issues, such as qualitative data and our systematic literature review [23]. Furthermore, we retrieved many issues from different perspectives (e.g., cancer survivors, OOPs, employers) in this extensive overview of QWL issues and therefore achieved a wide breadth of coverage.

The qualitative research methods were suitable for the purpose of generating QWL issues. In the focus groups, cancer survivors and OOPs clarified themselves and asked each other questions, which generated more outcomes than if all participants had been interviewed independently [25]. Although this is an advantage of a focus group, we chose to conduct interviews with employers, supervisors, and organization officers because the success of a focus group depends largely on the input of the participants, and if the topic is too personal or carried out in an institutional context, participants might feel reluctant to speak freely [26]. Because the topic of our focus group might have discredited a participant’s organization, for example a negative encounter with a sick employee, this could prevent them from feeling safe and from sharing their experiences within the group. That, in turn, would diminish the quality of the outcomes, and therefore, we presumed that the interviews were more appropriate for the sample with the employers, supervisors, and organization officers.

A key concern about the selection of QWL issues is due to the sample of cancer survivors in this study. The sample of cancer survivors that participated in the focus groups and to whom we administered the online questionnaire was moderately to highly educated and performed mainly white-collar jobs. This was not unexpected, as earlier research indicated that participants who participate in research often differ in socioeconomic variables compared to the nonresponsive participants, in particular level of education [27, 28]. However, when we take into consideration the social class differences between white-collar workers and blue-collar workers, the latter experience more repetitive work, lower skill discretion, lower job influence, higher job insecurity, and more environmental exposures [29]. In this study, having well-educated participants might have influenced the results in such a way that important QWL issues of blue-collar workers were missed. However, two comments on this assumption can be made. First, in the process of generating issues, we also included OOPs and employers who had experiences with supporting employees with cancer diagnoses in different work sectors. Therefore, QWL issues that could be relevant to workers in blue-collar jobs were most probably generated in the focus groups and interviews during this study. Secondly, we combined the issues from the focus groups and interviews with the literature review [23], which is based on 61 scientific articles, and then presented the issues for selection. However, we might not have prevented some QWL issues from being rated as irrelevant by our sample, while in fact, they were indeed relevant for workers in blue-collar jobs.

### QWL issues

Due to the subjective angle of the definition of QWL, “the experiences and perceptions of a cancer survivor in the work environment”, we would assume that asking cancer survivors directly about their QWL would lead to more issues containing specific feelings and lesser issues about job or organizational characteristics such as “work hours” or “communication within the organization”. In line with our expectations, cancer survivors did report multiple issues that included feelings, such as “doing what you like” or “being appreciated” or “feeling safe and familiar at the workplace” or “have meaning to the organization and society”. Something that was less expected was that the interviews with the employers, supervisors, and other organization officers also generated many issues about the feelings of cancer survivors. By including the employers, supervisors, and other organization officers, we therefore generated more relevant QWL issues about the individual work perception of cancer survivors.

After analyzing the outcomes of the online questionnaire, 44 issues were excluded from the initial questionnaire because they were rated as not relevant. Some were unexpected, for instance “discrimination and stigmas at work”, “an over-concerned social environment”, and “negative reactions from colleagues”. While previous research reported discrimination in the workplace as a problem for cancer survivors [30–34], in this study, it was rated as irrelevant for the QWL of working cancer survivors. The articles that discussed discrimination did not include studies that were conducted in the Netherlands; instead, the studies were done in the USA, UK, Canada, Sweden, or New Zealand. The exclusion of the issue “discrimination and stigmas at work” does not diminish the usefulness of the QWLQ-CS, it indicates that this version has been developed for the Dutch cancer population, and for future usage, the wording of the issues might be adjusted to the country of interest.

Our emphasis on the experiences and perceptions of a cancer survivor in the work environment is different from existing questionnaires that examine the quality of working life of healthy employees [16, 18], healthcare professionals in general [15], or nurses [20]. For instance, items of the Work-Related Quality of Life scale (WRQoL Scale) [15] stated “I am satisfied…”, “I feel well…”, and “I have been feeling reasonable happy…”. We presume that cancer survivors experience more specific feelings than these generic statements while dealing with changing work situations, and emotional and physical difficulties [33]. This assumption is supported by cancer survivors in the focus groups that reported issues about emotions such as being valued and feelings of vulnerability or helplessness. This initial version of the QWLQ-CS is therefore different from current QWL questionnaires, because it does not only contain generic items such as “I am happy with my work” but also covers more issues about the individual work perceptions. Furthermore, the existing questionnaires were not developed for cancer survivors and did not contain disease-specific items about the effect of the disease and the treatment. This category and others, for example “social structure and environment”, contain items that are specific for cancer survivors. Items such as “my supervisor understands my disease and its symptoms”, or “I disclose my disease and the current situation” are both disease-specific items. Overall, we presented a more in-depth approach to QWL of cancer survivors and more disease-specific items than previous QWL questionnaires did.

It is noteworthy to recognize the strengths and limitations of a disease-specific instrument. One limitation is the generalizability of the questionnaire; the outcomes on the QWLQ-CS cannot be compared with different working patient populations. One strength is that the content validity of the QWLQ-CS, with regard to cancer survivors, is probably higher than a generic instrument, as the items are found to be relevant to this specific population. Another strength is that the outcomes on disease-specific instruments can guide to more specific interventions that target the particular problems that cancer survivors face at the workplace. Previous research also implies that disease-specific instruments might be more responsive to changes than generic instruments [35, 36]. In sum, the choice of developing a generic or disease-specific questionnaire depends on the goal of the instrument. We decided to develop a disease-specific questionnaire, as our goal is to evaluate specifically the QWL of cancer survivors.

### Practical relevance

Although implementation of the QWLQ-CS should await further testing of its validity, reliability, and responsiveness, this initial version of the QWLQ-CS provides an overview of the multiple issues that contribute to the QWL of cancer survivors. For instance, it provides information about the experiences and perceptions of a cancer survivor in the work environment. Therefore, it can be useful in increasing the awareness of the potential problems or emotional difficulties working cancer survivor’s face. This awareness might lead to more support from OOPs, employers, supervisors, and other organization officers during the return-to-work and work continuation process of cancer survivors.

### Implication for research

As this study was a phase between having no questionnaire at all and a well-tested questionnaire, the next phase in the guidelines for developing Questionnaire Modules of the EORTC Quality of Life Group [21] is to perform a pretest study in which the initial QWLQ-CS will be tested on relevance, acceptability, and comprehensiveness. Afterward, the psychometric properties (e.g., validity, reliability, and responsiveness) of the questionnaire will be tested in a field study. Special attention will be paid to the length of the QWLQ-CS, as a long (online) questionnaire is associated with lower participation rates, and it is suggested that items placed at the end of the questionnaire produce lower quality data [37]. The objective of the definite QWLQ-CS is to use it for evaluative purposes, such as to measure the success of existing or new interventions that aims to improve QWL. However, future studies might consider how to develop interventions that improve the QWL of working cancer survivors based on the QWL issues from this study. Such interventions could be developed as part of support programs during the return-to-work and work continuation process.

## Conclusion

This initial version of the Quality of Working Life Questionnaire for Cancer Survivors (QWLQ-CS) is, to the best of our knowledge, the first questionnaire that evaluates the quality of working life of cancer survivors. Its 102 items cover the experiences and perceptions of a cancer survivor in the work environment. This initial version may increase awareness of the potential problems or emotional difficulties working cancer survivors face during the return-to-work or work continuation process; however, future research needs to pretest and examine the psychometric properties of the QWLQ-CS.
